# Applicability of optical coherence tomography angiography (OCTA) imaging in Parkinson’s disease

**DOI:** 10.1038/s41598-021-84862-x

**Published:** 2021-03-09

**Authors:** Jost L. Lauermann, Jan A. M. Sochurek, Pauline Plöttner, Florian Alten, Meike Kasten, Jannik Prasuhn, Norbert Brüggemann, Mahdy Ranjbar

**Affiliations:** 1 Department of Ophthalmology, University of Münster, Münster, Germany; 2 Laboratory for Angiogenesis and Ocular Cell Transplantation, University of Lübeck, Ratzeburger Allee 160, 23538 Lübeck, Germany; 3 Department of Neurology, University of Lübeck, Lübeck, Germany; 4 Institute of Neurogenetics, University of Lübeck, Lübeck, Germany; 5 Department of Psychiatry and Psychotherapy, University of Lübeck, Lübeck, Germany; 6 Department of Ophthalmology, University of Lübeck, Lübeck, Germany

**Keywords:** Parkinson's disease, Neurodegenerative diseases, Parkinson's disease

## Abstract

To evaluate the significance of motion artifacts in optical coherence tomography angiography (OCTA) images of patients with Parkinson’s disease (PD) and healthy controls. In this prospective, cross-sectional study subjects with medicated PD (ON) and healthy, age- and gender-matched volunteers were recruited. Participants underwent specific ophthalmological examinations, including OCTA. Angiograms of the superficial retinal capillary plexus were evaluated for the type and frequency of artifacts using a validated motion artifact score (MAS). A total of 30 PD patients (60 eyes), average disease duration of 9.61 ± 5.55 years, and 30 matched, healthy controls (60 eyes) were recruited. Twenty percent of all eyes had an eye disease, unknown to the participant, with a significant impact on OCTA results. After cleansing the dataset by excluding subjects with confounding ocular comorbidities 42 eyes of 28 PD patients and 53 eyes of 29 healthy controls were further evaluated. Overall MAS and all five subtypes of motion artifacts were comparable without significant differences between groups. OCTA can be used in treated PD patients (ON) without a significant increase in motion artifacts. Nevertheless, special attention should be paid to image quality during the acquisition of OCTA data, for which an experienced OCTA operator is useful.

## Introduction

The retina is part of the central nervous system and contains dopaminergic amacrine cells within the inner nuclear layer^1^. Interestingly, retinal dopamine and its metabolites were found to be significantly lower in Parkinson’s disease (PD)^2^. Besides, PD-associated microangiopathy contributing to clinical onset and progression of PD has been identified^3^.

With the advent of first-generation optical coherence tomography (OCT) devices, a new era of retinal imaging began, which also impacted other medical disciplines like neurology as more and more retina-associated biomarkers became the focus of interdisciplinary research^4^. OCT studies demonstrated significant thinning of the inner retinal layers in PD^5,6^. Correspondingly, electrophysiological studies provided evidence of retinal impairment in patients affected by PD^7^.

OCT angiography (OCTA) represents the latest evolution in OCT technology, a non-invasive, depth-selective, and highly reproducible modality that allows a three-dimensional mapping of the microvasculature^8^. OCTA of the retina and choroid has been proven to be a highly sensitive tool to detect microvascular abnormalities in ocular, but also systemic diseases^9–12^. Several OCTA studies reported on Alzheimer’s disease and found a reduced retinal flow density^13,14^. However, corresponding data on PD is sparse, as to date, only two groups reported on retinal microvascular abnormalities in PD patients using OCTA^15,16^.

Notably, caution is warranted when interpreting quantitative OCTA data. OCTA is a motion-contrast technique based on changes in successively recorded images. Consequently, examinations are susceptible to axial and transverse eye motion, which still represents one of the major sources of image artifacts^17,18^. OCTA hardware and software algorithms are both continuously developed further to compensate for movement artifacts. Yet, particularly in patients with low fixation due to eye diseases or movement disorders such as PD, OCTA imaging can be challenging, and image quality is frequently an issue. At the same time, however, OCTA image quality is of decisive importance for reliable quantitative image evaluations, especially when automated in-device analyzation tools are used^19^.

Therefore, this prospective study aimed to evaluate the applicability of OCTA in PD patients and to assess the prevalence and type of motion artifacts in comparison to healthy controls.

## Methods

### Participants

Participants were selected from a large epidemiological, longitudinal study on nonmotor symptoms in PD, the EPIPARK study (Epidemiology of nonmotor symptoms in Parkinson’s disease: frequency, characteristics, specificity, and course)^20^. All participants were selected according to the following exclusion criteria: age < 18 years, a high-grade visual loss, known macular disease or glaucoma, history of intraocular surgery (all participants), evidence for atypical or secondary parkinsonism, concurrent neurodegenerative diseases, known structural brain lesions, Hoehn and Yahr stage 5 (PD patients), and any neurological disease (controls). Invitation letters with detailed information of this sub-study were sent to potentially eligible subjects between January and February 2017. After one to two weeks, as announced in the letter, participants were contacted by phone, unless a study participation was rejected prior to the call, to reevaluate exclusion criteria and discuss further details such as an appointment.

Experienced movement disorder specialists performed the clinical examination. The patients took their regular anti-parkinsonian medication and were examined in the ON medication state. The motor evaluation was based on the Movement Disorder Society Unified Parkinson's Disease Rating Scale motor part (MDS-UPDRS-III). All participants underwent extended examinations including the assessment of blood pressure (BP), refraction, best-corrected visual acuity (BCVA) in Snellen, intraocular pressure (IOP), axial length (AL), and slit-lamp biomicroscopy including indirect fundoscopy by an experienced ophthalmologist. Only individuals without ocular complaints, except for the use of glasses, and no history of prior ophthalmic surgery were included in this study. Furthermore, the maximum permissible spherical aberration was ± 3 diopters. All participants and/or their legal guardians gave written informed consent before study enrollment. The study was carried out in accordance with relevant guidelines as well as regulations and was approved by the ethics committee of the University of Lübeck (AZ15-082).

### Imaging and image analysis

**Figure 1:**
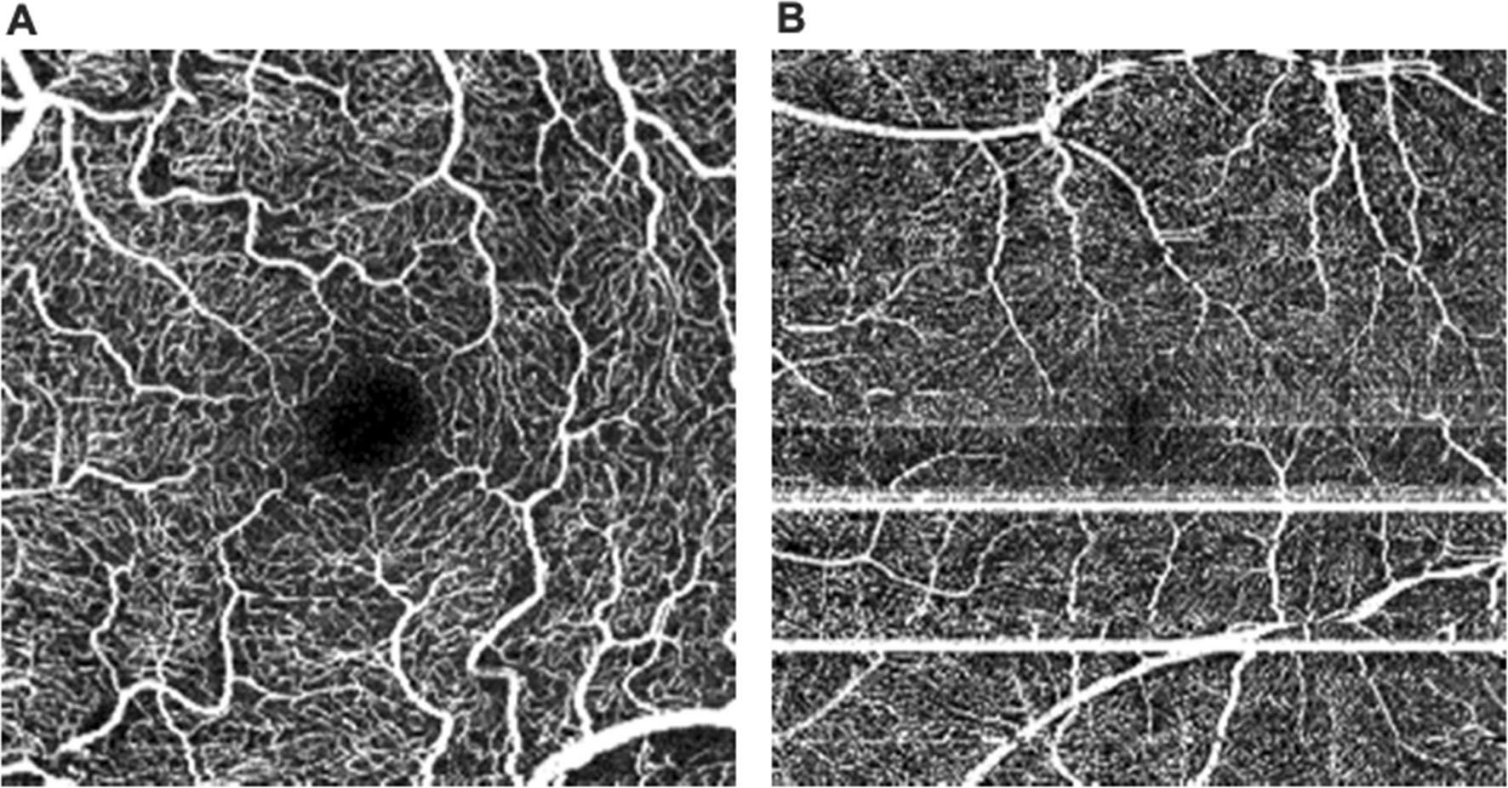
The motion artifact score (MAS) ranges from 1 to 4. While a MAS of 1 (**A**) indicates no or only slight banding/quilting and absence of all other artifacts due to motion or software correction, a MAS of 4 (**B**) implies significant banding/quilting in more than two quadrants, displacement in more than two quadrants, vessel doubling in more than two quadrants, stretch artifacts in more than two quadrants, or significant black line (Supplemental Table S1). While the former image (**A**) can be used for quantitative analysis of OCTA metrics, the latter one (**B**) should be excluded.

All images were captured under standardized conditions without prior pupil dilatation by a single medical student in the advanced course of studies, who received detailed instructions on how to operate the OCTA device (HS100, Angio eXpert, OCTA Version 2.0; Canon, Tokyo, Japan). This device uses a modified full-spectrum amplitude decorrelation algorithm (FSADA) to generate angiograms and offers active eye-tracking. Up to five volumetric OCTA (3 × 3 mm^2^) scans of the macula were obtained, and the scan with the highest signal strength was chosen for further analyses. The corresponding angiogram of the superficial retinal capillary plexus (SRCP) was automatically segmented from the inner limiting membrane (ILM) to 50 μm above the ganglion cell layer (GCL) by the device-own software^21^. Each angiogram was then exported and pseudonymized, before being evaluated concerning image quality by two independent, experienced OCTA artifact readers. Parameters for the evaluation of OCTA image quality were the expression of specific motion artifacts, i.e., banding/quilting, blink lines, displacement, stretching, and vessel doubling, following the OCTA motion artifact score (MAS, Supplemental Table S1) that has been previously described in detail (Fig. 1)^18,22^. Since the comparability of MAS is enhanced by centration of the OCTA image on the fovea and clear optic media, these additional parameters, centration (1: not perfectly centered, 2: perfectly centered) and vitreous opacities (1: present, 2: not present) were also assessed by the two readers. In a final step, both readers defined the presence of each of the five specific motion artifacts together in every single image. In case of any discrepancy, the evaluation of the senior reader was selected.

### Statistical analysis

The data were analyzed using IBM SPSS (Version 24.0, Chicago, IL, USA). Snellen BCVA was converted to the logarithm of the minimum angle of resolution (logMAR). The data were tested for normality using the Shapiro–Wilk test. To compare the means of the PD and control group, an independent-samples t-test (continuous variables) or a chi-square test (categorical) was conducted. In addition, predictors of MAS were evaluated by multiple regression analysis. Values are expressed as mean ± standard deviation and a *p* value of < 0.05 was considered statistically significant.

### Informed consent

Written informed consent was obtained from each subject and/or their legal guardians before enrollment in the study.

## Results

A total of 30 PD patients (60 eyes) and 30 healthy, age- and gender-matched controls (60 eyes) were recruited. Demographics and clinical data of the enrolled subjects are shown in Table 1. There were no statistical differences in the age and distribution of gender between PD patients and healthy controls. The comparison of axial length (AL), best-corrected visual acuity (BCVA), intraocular pressure (IOP), and mean arterial pressure (MAP) was not statistically significantly different between groups (*p* > 0.05). The average duration of the disease was 9.61 ± 5.55 (range 1–24) years in patients with PD, and their Hoehn and Yahr, as well as MDS-UPDRS-III scores, were 1.56 ± 0.93 and 24.26 ± 12.32, respectively.

**Table 1:** Demographic and clinical data.

	PD group (60 eyes)	Control group (60 eyes)	*p*
Age (years)	69.90 ± 8.16 (51–86)	69.97 ± 9.45 (48–83)	0.967
Gender (F/M)	15/15	14/16	0.715
MAP (mmHg)	94.45 ± 6.83	96.38 ± 7.74	0.453
Disease duration (years)	9.61 ± 5.55 (1–24)	N/A	–
Hoehn and Yahr scale	1.56 ± 0.93	0.0 ± 0.0	–
MDS-UPDRS-III score (ON)	24.26 ± 12.32	2.83 ± 3.34	–
BCVA (logMAR)	0.09 ± 0.13	0.05 ± 0.08	0.118
IOP (mmHg)	15.44 ± 3.08	16.12 ± 3.26	0.262
AL (mm)	23.63 ± 1.53	23.98 ± 0.91	0.415

*AL* axial length, *BCVA* best-corrected visual acuity, *F* female, *IOP* intraocular pressure, *logMAR* logarithm of the minimal angle of resolution, *M* male, *N/A* not applicable, *MDS-UPDRS* Movement Disorder Society Unified Parkinson's Disease Rating Scale; Results are reported as mean ± standard deviation (range). *p* < 0.05 was considered statistically significant.

Image quality results are reported in Table 2. Evaluation of OCTA image quality did not show any significant differences in signal strength and centration on the fovea (*p* > 0.05) between groups. A very high degree of reliability was found between MAS measurements of both graders. The average measure ICC was 0.99, with a 95% confidence interval from 0.985 to 0.993 (*p* < 0.001). However, MAS showed no significant difference between groups. Of all five MAS elements only “displacement” was significantly more often in PD patients compared to healthy controls (*p* = 0.036). Moreover, opacities of the vitreous were more frequently in controls (*p* = 0.008).

**Table 2:** Image quality assessment.

	PD group (60 eyes)	Control group (60 eyes)	*p*
Signal strength	6.38 ± 1.12	6.15 ± 1.19	0.303
MAS	3.11 ± 1.08	2.89 ± 1.17	0.285
Banding/quilting	90%	88%	0.846
Black line	47%	45%	0.799
Vessel doubling	3%	3%	0.165
Stretching	68%	53%	0.071
Displacement	25%	10%	**0.036**
Perfect centration	85%	90%	0.500
Vitreous opacities	0%	12%	**0.008**

*MAS* motion artifact score; Results are reported as mean ± standard deviation (range) or as percentage of affected images. *p* < 0.05 was considered statistically significant and bold font indicates statistical significance.

After cleansing the data for ophthalmologic comorbidities, which might have had an impact on MAS and OCTA results, 42 eyes of 28 PD patients and 53 eyes of 29 healthy controls remained. In PD patients, nine eyes (15%) with a clinically significant cataract, eight eyes (13.3%) with an epiretinal membrane (ERM), and one eye (1.7%) with dry age-related macular degeneration (AMD) had to be excluded. In the control group, two eyes (3.3%) with vitreomacular traction (VMT), two eyes (3.3%) with degenerative myopia (DM), one eye (1.7%) with AMD, one eye (1.7%) with a clinically significant cataract, and another one (1.7%) with an ERM had to be ignored. The prevalence of cataract (*p* = 0.008) and ERM (*p* = 0.015) were significantly higher in the PD group compared to the control group.

Demographics and clinical data of the selected subgroup are listed in Table 3. The data cleansing resulted in an even more pronounced alignment of image quality between groups. Overall MAS and all five elements did not show significant differences any longer (Table 4). However, vitreous opacities were still predominantly found in healthy controls (*p* = 0.025).

**Table 3:** Demographic and clinical data of the selected subgroup.

	PD group (42 eyes)	Control group (53 eyes)	*p*
Age (years)	69.67 ± 9.16 (51–86)	69.61 ± 9.55 (48–83)	0.982
Gender (F/M)	14/14	14/15	0.898
MAP (mmHg)	94.79 ± 6.72	95.96 ± 7.59	0.551
Disease duration (years)	9.74 ± 6.15 (1–24)	N/A	–
Hoehn and Yahr scale	1.47 ± 0.90	0.0 ± 0.0	–
MDS-UPDRS-III score (ON)	24.32 ± 10.95	2.57 ± 3.24	–
BCVA (logMAR)	0.03 ± 0.06	0.03 ± 0.05	0.955
IOP (mmHg)	15.42 ± 2.90	16.57 ± 3.04	0.078
AL (mm)	23.65 ± 1.66	23.96 ± 0.93	0.510

*AL* axial length, *BCVA* best-corrected visual acuity, *F* female, *IOP* intraocular pressure, *logMAR* logarithm of the minimal angle of resolution, *M* male, *N/A* not applicable, *MDS-UPDRS* Movement Disorder Society Unified Parkinson's Disease Rating Scale; Results are reported as mean ± standard deviation (range). *p* < 0.05 was considered statistically significant.

**Table 4:** Image quality assessment of the selected subgroup.

	PD group (42 eyes)	Control group (53 eyes)	*p*
Signal strength	6.49 ± 1.16	6.23 ± 1.21	0.303
MAS	3.04 ± 1.17	2.78 ± 1.18	0.296
Banding/quilting	86%	87%	0.871
Black line	48%	42%	0.697
Vessel doubling	0%	2%	0.372
Stretching	62%	49%	0.215
Displacement	19%	9%	0.172
Perfect centration	88%	89%	0.922
Vitreous opacities	0%	11%	**0.025**

*MAS* motion artifact score; Results are reported as mean ± standard deviation (range) or as percentage of affected images. *p* < 0.05 was considered statistically significant and bold font indicates statistical significance.

A multiple regression analysis was carried out to investigate whether MDS-UPDRS-III and gaining experience as an OCTA operator over time could significantly predict the occurrence of motion artifacts as registered by MAS. The results of the regression analysis indicated that in PD patients the model explained 33.1% of the variance and that the model was a significant predictor of motion artifact occurrence, F(2,33) = 8.16, *p* = 0.001. While MDS-UPDRS-III (B = 0.065, *p* < 0.001) contributed significantly to the model, gaining experience as an OCTA operator (B = 0.003, *p* = 0.680) did not. In healthy subjects the model explained 27.1% of the variance and it was a significant predictor of motion artifact occurrence, F(2,49) = 9.10, *p* < 0.001. Once again, MDS-UPDRS-III (B = 0.173, *p* < 0.001) contributed significantly to the model, however, gaining experience as an OCTA operator (B = −0.010, *p* = 0.017) did also now. Finally, the multiple regression analysis was repeated in the PD cohort after adding disease duration as an independent variable to the mix. The results suggested that this extended model explained 42.4% of the variance and that the model was a significant predictor of motion artifact occurrence, F(3,32) = 7.84, *p* < 0.001. MDS-UPDRS-III (B = 0.060, *p* < 0.001) and disease duration (B = 0.061, *p* = 0.030) contributed significantly to the model, while gaining experience as OCTA operator (B = 0.003, *p* = 0.652) did not.

## Discussion

The present study demonstrates that motion artifacts in OCTA images are equally common in medicated patients with PD (ON) and age- and gender-matched, healthy controls. Longer disease duration and more severe motor symptoms, however, were associated with a higher degree of motion artifacts in PD. Interestingly, a similar tendency was also observed in controls at slight motor impairment due to non-PD-related causes. Thus, caution and a certain level of expertise is needed during the evaluation of outputted images with regard to quality and the interpretation of automatically generated results, at least in PD patients at more advanced stages of the disease.

OCTA offers great potential not just for ophthalmological issues, but also presents a new diagnostic tool in systemic diseases as several studies have already indicated a benefit of OCTA and potentially new OCTA-derived biomarkers in primary, non-ophthalmic diseases^10,23^.

In 2018, Kwapong and colleagues published the first study examining PD patients using OCTA^15^. In that study the authors focused on finding potential vascular biomarkers based on quantitative measurements. They described a reduced retinal microvascular density in PD patients as well as a correlation between microvascular impairment and inner retinal thinning. Recently, Robbins and co-workers confirmed these OCTA findings^16^. Apart from this work, previous studies on the retina in PD patients were exclusively based on structural OCT scans. With OCT technology, one could already analyze morphological changes in different neurological diseases. However, OCTA will significantly expand the diagnostic possibilities here because now the functional, vascular component can also be examined more closely.

Despite these appealing perspectives, there are important limitations. Kwapong and colleagues reported that at least six patients with PD and two healthy controls had to be excluded due to motion artifacts, which underlines the relevance of the present study^15^. It is known that artifacts and in particular artifacts due to motion also occur during OCTA imaging in healthy subjects, as confirmed in the present study. In contrast to segmentation errors, which show disease-specific differences, the occurrence of motion artifacts mainly depends on the compliance of the patient, which is not only due to ophthalmological diseases but also due to systemic diseases, increased age, or possibly low motivation to cooperate during the investigation^18,24^. In our study, motor disability due to PD or other causes was found to be an important positive predictor for the occurrence of motion artifacts.

PD is characterized by cardinal motor symptoms like tremor, bradykinesia, and rigidity, as well as neuropsychiatric symptoms like a reduced control of attention or bradyphrenia^25^. Moreover, the motor symptoms of PD become progressively worse as the disease advances^26^. In accordance with this, motion artifacts increased with duration of PD in our study population. These factors would suggest that obtaining OCTA recordings free from motion artifacts in PD patients could be quite challenging, especially in later stages of the disease. This might be the reason why Kwapong et al. focused on patients in early stages of PD^15^.

Interestingly, however, the present work does not show a higher number of motion artifacts in patients with PD compared to healthy subjects. Patients seemed to be able to cooperate as well as healthy individuals for the duration of the examination. Microsaccades and minute eye movements in healthy subjects, possibly due to a lack of concentration, thus seem to be just as problematic as a potentially more considerable motor agitation and likely reduced concentration in PD. On top of that, it is also quite important to highlight that in the study of Kwapong et al. as well as ours all patients took their medication on a regular basis and were thus in the ON state^15^. With regard to motion artifacts, results can be expected to be much higher in patients without medication (OFF).

Despite comparable results concerning the total frequency of motion artifacts in both groups, it is noteworthy, that motion artifacts were also clearly higher in our control group compared to the literature^18,22,27^. One possible explanation could be the experience of the user who took the OCTA images. In this study, those were not performed by a retinal specialist with expertise in OCTA imaging. It may be presumed that experience and training in operating the device are key for improving the quality of recordings. Indeed, our results suggest that gaining experience in OCTA imaging is a negative predictor for the occurrence of motion artifacts as registered by MAS, at least in subjects without PD. The more OCTA examinations our single operator performed over time the fewer motion artifacts were detected by our two specialized readers, and henceforth the lower MAS.

In context of our generally quite high MAS readings, it is of interest that MAS was found to be lower during mydriatic examinations, especially in the elderly^28^. This could be at least considered when focusing on quantitative OCTA analyses in PD patients, but needs to be weighed up against the risk of accidents due to blurry vision, especially in a motorically handicapped cohort. Interestingly, the status of the pupil during examinations was neither reported by Kwapong and colleagues nor Robbins and co-workers^15,16^.

We assume that at most centers, clinical practice is that OCT and OCTA images are not taken by ophthalmologists, but by properly trained non-medical staff. Woetzel et al. discussed the influence of different expertise levels in OCTA image quality assessment^22^. They concluded that trained non-ophthalmologic assessors could perform OCTA image quality assessment in a sufficient manner. However, it is most reliably performed by an ophthalmologist with knowledge in retinal image analysis. This need is also underlined by the fact that a total of 25 eyes (20.8%) in our study had a disease, so far unknown to the patient, but potentially impacting OCTA flow density results^18,29,30^. Actually, ophthalmologic disorders appeared to be more common in our PD cohort. Visual disorders like double vision, dry eyes, visual field deficits and cataract are common in PD^31,32^. We also found significant more cataracts in the PD group than in matched controls. Additionally, the prevalence of ERM in PD was substantially higher, which could be due to inner retinal alterations associated with PD, but has not been reported, so far. However, this is at most a preliminary finding that doesn’t allow for any generalized conclusion and needs to be evaluated in larger epidemiological studies. Moreover, there is a likely biased coincidence as ophthalmological research tends to attract participants who are concerned about their vision and in need of a check-up.

OCTA recordings are very complex, both in terms of their data volumes and their potential significance as well as the presence of error sources. Automated in-device calculations and the unfiltered, immediately generated results should only be considered cautiously. In comparison, MRI scans are assessed by specialized radiologists or neuroradiologists to provide the most reliable basis for colleagues from other departments for clinical interpretation of the data. It would be conceivable that specialized eye clinics serve to create and evaluate OCTA recordings and then make the revised results available to non-specialist colleagues for clinical use.

At the same time, it can be assumed that further technical improvements will facilitate the creation of high-quality recordings in the future. In addition to eye tracking, recently, new features such as multiple image averaging and deep learning-based intelligent denoise were introduced to assist the user^27^.

There are several limitations to the current study. The images were taken with only one single OCTA device. However, this device is commercially available and can be compared to other OCTA devices based on its technical characteristics. Moreover, no a priori power analysis took place to assess an appropriate sample size for this study and it can be argued that the sample size of our study might be too small to discriminate MAS differences. Yet, the sample size it is still within the range of previous MAS studies. Furthermore, there is very little data on the use of OCTA in PD, and this is also the first work explicitly dealing with motion artifacts in this context. It would be desirable for future OCTA studies in PD to evaluate the results obtained here on a larger scale.

In summary, OCTA can be used in patients with severe neurological diseases such as PD without any significant loss of image quality due to motion artifacts compared to healthy subjects. Nevertheless, motion artifacts remain a common problem in OCTA imaging. Users must take this into account, rule out erroneous recordings, and repeat them if necessary. Interdisciplinary cooperation between ophthalmologists and other specialists, e.g., neurologists, will be the basis for the most comprehensive, and at the same time most reliable applications in the future. Specialized reading centers could also contribute to a highly reliable evaluation of OCTA recordings in clinical studies.

In the future, technical advancements will further improve OCTA images. So far, exploiting the full potential of the technology requires a critical evaluation of the imaging quality and appropriate experience with the technology. If this is the case, OCTA appears to be an exciting new tool to gain new insights into disease progression from microperfusion changes in PD.

## Supplementary information

Supplementary Table S1.
