# Supplementary material for: Applicability of optical coherence tomography angiography (OCTA) imaging in Parkinson’s disease
Source: Sci Rep. 2021 Mar 9;11:5520. doi: 10.1038/s41598-021-84862-x (PMC7943590; doi:10.1038/s41598-021-84862-x)
Supplement: Supplementary file 1 — Supplementary Table S1. [file 41598_2021_84862_MOESM1_ESM.docx]

**Applicability of Optical Coherence Tomography**

**Angiography (OCTA) Imaging in Parkinson’s Disease**

Jost L. Lauermann ^1,†^, Jan A. M. Sochurek ^2,†^, Pauline Plöttner ^3^, Florian Alten ^1^,

Meike Kasten ^4,5^, Jannik Prasuhn ^3,4^, Norbert Brüggemann ^3,4^, and Mahdy Ranjbar ^2,6,*^

1) Department of Ophthalmology, University of Münster

2) Laboratory for Angiogenesis and Ocular Cell Transplantation, University of Lübeck

3) Department of Neurology, University of Lübeck

4) Institute of Neurogenetics, University of Lübeck

5) Department of Psychiatry and Psychotherapy, University of Lübeck

6) Department of Ophthalmology, University of Lübeck

†) JLL and JAMS contributed equally and should be regarded as equivalent first authors

*) Corresponding author:

M. Ranjbar, Ratzeburger Allee 160, 23538 Lübeck, Germany

Phone: +49-451-500-43913

Fax: +49-451-500-43914

Email: eye.research101@gmail.com

**Disclosures**

1) Conflict of interest: None.

2) Consent: Written informed consent was obtained from each subject and/or their legal guardians before enrollment in the study.

3) Funding/Support: None.

4) Financial Disclosures: None.

**Supplemental**

Supplemental Table S1: Motion artifact score (MAS)

| **Score** | **Definition** |
| --- | --- |
| 1 | Required: no or slight banding/quilting, absence of all other artifacts due to motion or software correction |
| 2 | Required: slight or moderate banding/quilting, non-significant black line |
| 3 | Required: moderate banding/quilting or significant banding/quilting in one or two quadrants, displacement in one or two quadrants, vessel doubling in one or two quadrants, stretch artifacts in one or two quadrants, non-significant black line |
| 4 | Required: significant banding/quilting in more than two quadrants, displacement in more than two quadrants, vessel doubling in more than two quadrants, stretch artifacts in more than two quadrants, significant black line |
